# 5-Butyl­amino-2-[2-(dimethyl­amino)eth­yl]-1*H*-benz[*de*]isoquinoline-1,3(2*H*)-dione

**DOI:** 10.1107/S1600536810018702

**Published:** 2010-05-26

**Authors:** Li-Juan Xie

**Affiliations:** a Institute of Molecular Medicine, Huaqiao University, Quanzhou, Fujian 362021, People’s Republic of China.

## Abstract

The title compound, C_20_H_25_N_3_O_2_, is a new amonafide analogue, which exhibits anti­tumor activity. The asymmetric unit contains two mol­ecules with similar conformations for the substituted aliphatic chains. The two independent mol­ecules form dmers through N—H⋯N hydrogen bonds. The crystal structure is stabilized *via* π–π stacking inter­actions, the shortest centroid–centroid separation between six-membered rings being 3.673 (2) Å.

## Related literature

For general background to amonafide and its anti­tumour activity, see: Braña *et al.* (1981 ▶, 2001 ▶); Braña & Ramos (2001 ▶); Ratain *et al.* (1991 ▶, 1993 ▶). For the synthesis of amonafide analogues, see: Xie *et al.* (2009 ▶).
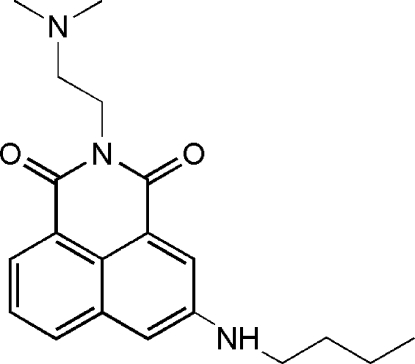


## Experimental

### 

#### Crystal data

C_20_H_25_N_3_O_2_
*M* *_r_* = 339.43
Triclinic, 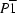

*a* = 11.5978 (12) Å
*b* = 12.5362 (13) Å
*c* = 14.3721 (16) Å
α = 72.329 (2)°
β = 70.599 (2)°
γ = 70.759 (2)°
*V* = 1816.1 (3) Å^3^
*Z* = 4
Mo *K*α radiation
μ = 0.08 mm^−1^
*T* = 293 K
0.36 × 0.33 × 0.08 mm

#### Data collection

Bruker SMART APEX CCD area-detector diffractometer
Absorption correction: multi-scan (*SADABS*; Bruker, 2001 ▶) *T*_min_ = 0.739, *T*_max_ = 1.000
10049 measured reflections
7018 independent reflections
3025 reflections with *I* > 2σ(*I*)
*R*_int_ = 0.059

#### Refinement

*R*[*F* ^2^ > 2σ(*F* ^2^)] = 0.068
*wR*(*F* ^2^) = 0.175
*S* = 0.86
7018 reflections
466 parameters
3 restraints
H atoms treated by a mixture of independent and constrained refinement
Δρ_max_ = 0.34 e Å^−3^
Δρ_min_ = −0.25 e Å^−3^

### 

Data collection: *SMART* (Bruker, 2001 ▶); cell refinement: *SAINT* (Bruker, 2001 ▶); data reduction: *SAINT*; program(s) used to solve structure: *SHELXS97* (Sheldrick, 2008 ▶); program(s) used to refine structure: *SHELXL97* (Sheldrick, 2008 ▶); molecular graphics: *SHELXTL* (Sheldrick, 2008 ▶); software used to prepare material for publication: *SHELXL97*.

## Supplementary Material

Crystal structure: contains datablocks global, I. DOI: 10.1107/S1600536810018702/bh2282sup1.cif

Structure factors: contains datablocks I. DOI: 10.1107/S1600536810018702/bh2282Isup2.hkl

Additional supplementary materials:  crystallographic information; 3D view; checkCIF report

## Figures and Tables

**Table 1:** Hydrogen-bond geometry (Å, °)

*D*—H⋯*A*	*D*—H	H⋯*A*	*D*⋯*A*	*D*—H⋯*A*
N2—H2⋯N6	0.86 (2)	2.33 (2)	3.176 (5)	173 (3)
N5—H5⋯N3	0.85 (2)	2.37 (2)	3.220 (5)	172 (4)

**Fig. 1.**
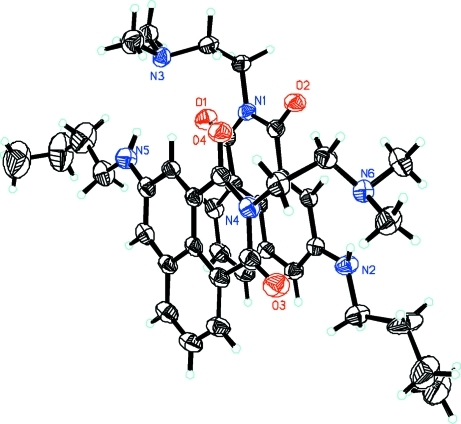
The structure of the title compound showing 30% probability displacement ellipsoids and the atom labeling scheme. H atoms are omitted for clarity.

**Fig. 2.**
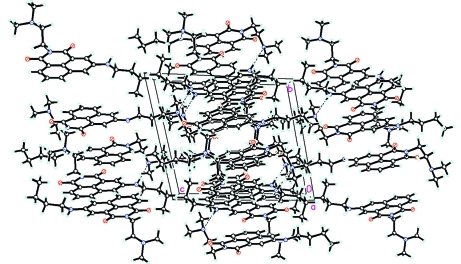
Packing diagram with H bonds indicated by dashed lines.

## supplementary crystallographic information

### Comment

Amonafide (Braña *et al.*, 1981, 2001; Braña & Ramos, 2001) was the first compound of the naphthalimide family that reached the clinical trial stage and exhibited excellent antitumour activity against advanced breast cancer. However, in the clinical studies, it was found that amonafide was easily metabolized to *N*-acetyl-amonafide by enzyme *N*-acetyltransferase, which caused a high-variable, unpredictable toxicity (Ratain *et al.*, 1991, 1993). In order to reduce the unpredictable toxic effect of the amonafide, we synthesized a series of amonafide analogues (Xie *et al.*, 2009) involved the title compound, which is being reported in this article.

The molecular structure of the title compound is shown in Fig. 1. The asymmetric unit contains two independent molecules, and it is observed that the butyl chains do not present the common all-*trans* conformation. This uncommon feature could be attributed to formation of intermolecular N—H···N hydrogen bonds in the asymmetric unit [N2··· N6 and N3···N5], which involve the butyl and the dimethylamino groups, and reduce the intermolecular hindrance. The crystal structure is stabilized *via π*-*π* stacking interactions [centroid-centroid separations: 3.673 (2) and 3.693 (2) Å] and intermolecular N—H···N hydrogen bonds, which lead to a supramolecular network of stacked molecules in 1D chains (Fig. 2). Apart from the functional groups butylamino and *N*,*N*-dimethylamino-ethylamino, the central 1,8-naphthalimide fused rings system is almost planar.

### Experimental

A mixture of 3-bromide-1,8-naphthalic anhydride (277 mg, 1.0 mmol) and *N*,*N*-dimethylethyldiamine (92 mg, 1.0 mmol) was refluxed in EtOH (15 ml) for 2 h, to give the intermediate 5-bromo-2-[2-(dimethylamino)ethyl]-1*H*-benz[*de*]isoquinoline-1,3(2*H*)-dione. This intermediate (174 mg, 0.5 mmol), CuI (9 mg, 0.05 mmol), proline (11 mg, 0.1 mmol), Cs_2_CO_3_ (244 mg, 0.75 mmol) and *n*-butylamine (0.75 mmol) in dry DMSO (2 ml) were mixed and stirred at 383 K for 9 h under nitrogen. The crude products were purified by chromatography on silica gel with a mixture of CH_2_Cl_2_ and MeOH as eluent. Single crystals of the title compound were obtained from a CH_2_Cl_2_—MeOH solution.

### Refinement

C-bonded H atoms were positioned geometrically and refined as riding atoms, with C—H = 0.93 (aromatic), 0.97 (methylene) or 0.96 Å (methyl). Isotropic displacement parameters were computed as *U*_iso_(H) = 1.2*U*_eq_(carrier C) for methylene and aromatic H atoms, and *U*_iso_(H) = 1.5*U*_eq_(carrier C) for methyl groups. Amine H atoms H2 and H5 were found in a difference map and refined freely.

### Crystal data

**Table:**

C_20_H_25_N_3_O_2_	*Z* = 4
*M**_r_* = 339.43	*F*(000) = 728
Triclinic, *P*1	*D*_x_ = 1.241 Mg m^−^^3^
Hall symbol: -P 1	Mo *K*α radiation, λ = 0.71073 Å
*a* = 11.5978 (12) Å	Cell parameters from 1325 reflections
*b* = 12.5362 (13) Å	θ = 5.2–49.0°
*c* = 14.3721 (16) Å	µ = 0.08 mm^−^^1^
α = 72.329 (2)°	*T* = 293 K
β = 70.599 (2)°	Prismatic, yellow
γ = 70.759 (2)°	0.36 × 0.33 × 0.08 mm
*V* = 1816.1 (3) Å^3^	

### Data collection

**Table:**

Bruker SMART APEX CCD area-detector diffractometer	7018 independent reflections
Radiation source: fine-focus sealed tube	3025 reflections with *I* > 2σ(*I*)
graphite	*R*_int_ = 0.059
φ and ω scans	θ_max_ = 26.0°, θ_min_ = 1.9°
Absorption correction: multi-scan (*SADABS*; Bruker, 2001)	*h* = −14→13
*T*_min_ = 0.739, *T*_max_ = 1.000	*k* = −15→11
10049 measured reflections	*l* = −17→16

### Refinement

**Table:**

Refinement on *F*^2^	Secondary atom site location: difference Fourier map
Least-squares matrix: full	Hydrogen site location: inferred from neighbouring sites
*R*[*F*^2^ > 2σ(*F*^2^)] = 0.068	H atoms treated by a mixture of independent and constrained refinement
*wR*(*F*^2^) = 0.175	*w* = 1/[σ^2^(*F*_o_^2^) + (0.0653*P*)^2^] where *P* = (*F*_o_^2^ + 2*F*_c_^2^)/3
*S* = 0.86	(Δ/σ)_max_ < 0.001
7018 reflections	Δρ_max_ = 0.34 e Å^−^^3^
466 parameters	Δρ_min_ = −0.25 e Å^−^^3^
3 restraints	Extinction correction: *SHELXL97* (Sheldrick, 2008), Fc^*^=kFc[1+0.001xFc^2^λ^3^/sin(2θ)]^-1/4^
0 constraints	Extinction coefficient: 0.0040 (10)
Primary atom site location: structure-invariant direct methods	

### Fractional atomic coordinates and isotropic or equivalent isotropic displacement parameters (Å^2^)

**Table:**

	*x*	*y*	*z*	*U*_iso_*/*U*_eq_	
O1	0.6424 (3)	0.8935 (2)	0.2061 (2)	0.0695 (9)	
O2	0.4583 (2)	0.7887 (2)	0.54144 (19)	0.0625 (8)	
O3	0.8585 (3)	0.6222 (3)	0.8127 (2)	0.0754 (9)	
O4	0.6754 (3)	0.5191 (3)	0.6307 (2)	0.0700 (9)	
N1	0.5553 (3)	0.8357 (3)	0.3739 (2)	0.0481 (8)	
N2	0.6845 (3)	0.9030 (3)	0.7247 (3)	0.0592 (10)	
N3	0.6185 (3)	0.6109 (3)	0.2983 (2)	0.0537 (9)	
N4	0.7643 (3)	0.5748 (3)	0.7207 (2)	0.0518 (8)	
N5	0.8927 (4)	0.6479 (3)	0.2640 (3)	0.0665 (11)	
N6	0.5323 (3)	0.7148 (3)	0.8571 (2)	0.0537 (9)	
C1	0.6412 (4)	0.8876 (3)	0.2925 (3)	0.0508 (10)	
C2	0.5400 (4)	0.8308 (3)	0.4763 (3)	0.0480 (10)	
C3	0.6272 (3)	0.8762 (3)	0.4991 (3)	0.0394 (9)	
C4	0.7179 (3)	0.9258 (3)	0.4200 (3)	0.0416 (9)	
C5	0.7264 (3)	0.9327 (3)	0.3189 (3)	0.0461 (10)	
C6	0.8156 (4)	0.9811 (3)	0.2421 (3)	0.0563 (11)	
H6	0.8212	0.9855	0.1750	0.068*	
C7	0.8974 (4)	1.0234 (4)	0.2660 (3)	0.0628 (12)	
H7	0.9573	1.0563	0.2144	0.075*	
C8	0.8902 (3)	1.0169 (3)	0.3642 (3)	0.0578 (11)	
H8	0.9458	1.0452	0.3784	0.069*	
C9	0.8004 (3)	0.9683 (3)	0.4447 (3)	0.0453 (10)	
C10	0.7902 (3)	0.9603 (3)	0.5474 (3)	0.0510 (10)	
H10	0.8439	0.9890	0.5635	0.061*	
C11	0.7023 (3)	0.9107 (3)	0.6237 (3)	0.0453 (10)	
C12	0.6205 (3)	0.8696 (3)	0.5963 (3)	0.0455 (10)	
H12	0.5599	0.8368	0.6472	0.055*	
C13	0.7608 (4)	0.9397 (4)	0.7643 (3)	0.0675 (13)	
H13A	0.7753	1.0137	0.7232	0.081*	
H13B	0.8422	0.8837	0.7618	0.081*	
C14	0.6929 (5)	0.9512 (5)	0.8749 (3)	0.108 (2)	
H14A	0.6063	0.9957	0.8775	0.130*	
H14B	0.6903	0.8744	0.9163	0.130*	
C15	0.7481 (6)	1.0049 (6)	0.9201 (4)	0.123 (2)	
H15A	0.8336	0.9585	0.9202	0.148*	
H15B	0.7003	1.0024	0.9901	0.148*	
C16	0.7529 (6)	1.1257 (5)	0.8708 (4)	0.129 (2)	
H16A	0.8098	1.1282	0.8046	0.194*	
H16B	0.7821	1.1557	0.9105	0.194*	
H16C	0.6700	1.1718	0.8654	0.194*	
C17	0.4680 (4)	0.7897 (3)	0.3524 (3)	0.0567 (11)	
H17A	0.4649	0.8224	0.2825	0.068*	
H17B	0.3838	0.8145	0.3948	0.068*	
C18	0.5044 (4)	0.6589 (3)	0.3698 (3)	0.0572 (11)	
H18A	0.5162	0.6266	0.4375	0.069*	
H18B	0.4349	0.6345	0.3667	0.069*	
C19	0.6511 (4)	0.4846 (4)	0.3333 (3)	0.0811 (14)	
H19A	0.5835	0.4551	0.3354	0.122*	
H19B	0.6640	0.4649	0.3997	0.122*	
H19C	0.7271	0.4513	0.2877	0.122*	
C20	0.5971 (4)	0.6400 (4)	0.1975 (3)	0.0798 (14)	
H20A	0.5241	0.6167	0.2020	0.120*	
H20B	0.6697	0.6004	0.1532	0.120*	
H20C	0.5835	0.7221	0.1714	0.120*	
C21	0.8560 (4)	0.6183 (4)	0.7304 (3)	0.0565 (11)	
C22	0.7540 (4)	0.5639 (3)	0.6306 (3)	0.0529 (11)	
C23	0.8405 (3)	0.6098 (3)	0.5360 (3)	0.0464 (10)	
C24	0.9353 (3)	0.6535 (3)	0.5412 (3)	0.0468 (10)	
C25	0.9461 (3)	0.6572 (3)	0.6348 (3)	0.0524 (11)	
C26	1.0414 (4)	0.6991 (4)	0.6382 (3)	0.0631 (12)	
H26	1.0481	0.7028	0.6998	0.076*	
C27	1.1271 (4)	0.7359 (4)	0.5481 (4)	0.0669 (13)	
H27	1.1913	0.7631	0.5505	0.080*	
C28	1.1178 (4)	0.7325 (4)	0.4568 (3)	0.0646 (12)	
H28	1.1761	0.7570	0.3981	0.078*	
C29	1.0213 (3)	0.6923 (3)	0.4503 (3)	0.0508 (10)	
C30	1.0069 (4)	0.6899 (3)	0.3573 (3)	0.0580 (11)	
H30	1.0636	0.7150	0.2976	0.070*	
C31	0.9108 (4)	0.6512 (3)	0.3526 (3)	0.0520 (10)	
C32	0.8288 (3)	0.6093 (3)	0.4460 (3)	0.0513 (10)	
H32	0.7651	0.5807	0.4447	0.062*	
C33	0.9693 (4)	0.6911 (4)	0.1663 (3)	0.0804 (15)	
H33A	1.0576	0.6533	0.1636	0.097*	
H33B	0.9576	0.7734	0.1576	0.097*	
C34	0.9377 (6)	0.6714 (5)	0.0831 (4)	0.119 (2)	
H34A	0.8494	0.7099	0.0861	0.142*	
H34B	0.9478	0.5891	0.0931	0.142*	
C35	1.0166 (7)	0.7137 (6)	−0.0212 (4)	0.157 (3)	
H35A	0.9636	0.7421	−0.0680	0.188*	
H35B	1.0436	0.7785	−0.0196	0.188*	
C36	1.1246 (7)	0.6296 (7)	−0.0586 (6)	0.207 (4)	
H36A	1.1944	0.6293	−0.0367	0.310*	
H36B	1.1453	0.6476	−0.1311	0.310*	
H36C	1.1075	0.5546	−0.0334	0.310*	
C37	0.6757 (4)	0.5292 (3)	0.8135 (3)	0.0606 (12)	
H37A	0.7080	0.5170	0.8711	0.073*	
H37B	0.6716	0.4546	0.8096	0.073*	
C38	0.5425 (4)	0.6093 (4)	0.8305 (3)	0.0581 (11)	
H38A	0.5146	0.6291	0.7694	0.070*	
H38B	0.4858	0.5677	0.8840	0.070*	
C39	0.4064 (4)	0.7929 (4)	0.8558 (3)	0.0811 (15)	
H39A	0.3898	0.8027	0.7921	0.122*	
H39B	0.4033	0.8667	0.8648	0.122*	
H39C	0.3438	0.7604	0.9095	0.122*	
C40	0.5512 (4)	0.6922 (4)	0.9577 (3)	0.0810 (15)	
H40A	0.5368	0.7644	0.9754	0.121*	
H40B	0.6362	0.6476	0.9576	0.121*	
H40C	0.4930	0.6499	1.0062	0.121*	
H2	0.649 (3)	0.850 (3)	0.763 (2)	0.065 (14)*	
H5	0.818 (3)	0.645 (4)	0.271 (3)	0.082 (17)*	

### Atomic displacement parameters (Å^2^)

**Table:**

	*U*^11^	*U*^22^	*U*^33^	*U*^12^	*U*^13^	*U*^23^
O1	0.087 (2)	0.081 (2)	0.0454 (18)	−0.0301 (18)	−0.0225 (16)	−0.0058 (15)
O2	0.0622 (18)	0.076 (2)	0.0559 (18)	−0.0390 (17)	−0.0017 (14)	−0.0159 (15)
O3	0.075 (2)	0.097 (3)	0.059 (2)	−0.0247 (19)	−0.0253 (17)	−0.0121 (17)
O4	0.0651 (19)	0.080 (2)	0.073 (2)	−0.0406 (18)	−0.0019 (15)	−0.0221 (16)
N1	0.0513 (19)	0.042 (2)	0.054 (2)	−0.0131 (16)	−0.0176 (16)	−0.0100 (16)
N2	0.070 (2)	0.063 (3)	0.052 (2)	−0.032 (2)	−0.0168 (19)	−0.0054 (19)
N3	0.058 (2)	0.054 (2)	0.053 (2)	−0.0076 (18)	−0.0205 (17)	−0.0165 (17)
N4	0.049 (2)	0.050 (2)	0.050 (2)	−0.0138 (17)	−0.0090 (16)	−0.0056 (16)
N5	0.068 (3)	0.079 (3)	0.051 (2)	−0.025 (2)	−0.010 (2)	−0.0106 (19)
N6	0.057 (2)	0.050 (2)	0.048 (2)	−0.0141 (18)	−0.0105 (16)	−0.0056 (16)
C1	0.055 (2)	0.042 (2)	0.051 (3)	−0.009 (2)	−0.016 (2)	−0.005 (2)
C2	0.048 (2)	0.040 (2)	0.054 (3)	−0.012 (2)	−0.011 (2)	−0.0103 (19)
C3	0.040 (2)	0.030 (2)	0.045 (2)	−0.0054 (17)	−0.0114 (17)	−0.0080 (17)
C4	0.040 (2)	0.031 (2)	0.047 (2)	−0.0052 (17)	−0.0103 (18)	−0.0046 (17)
C5	0.047 (2)	0.038 (2)	0.048 (2)	−0.0086 (19)	−0.0135 (19)	−0.0034 (18)
C6	0.057 (3)	0.057 (3)	0.044 (2)	−0.015 (2)	−0.009 (2)	−0.001 (2)
C7	0.051 (3)	0.063 (3)	0.063 (3)	−0.025 (2)	−0.006 (2)	0.004 (2)
C8	0.052 (3)	0.058 (3)	0.061 (3)	−0.022 (2)	−0.013 (2)	−0.003 (2)
C9	0.041 (2)	0.037 (2)	0.052 (2)	−0.0097 (18)	−0.0105 (19)	−0.0048 (18)
C10	0.051 (2)	0.045 (3)	0.061 (3)	−0.013 (2)	−0.020 (2)	−0.010 (2)
C11	0.048 (2)	0.040 (2)	0.047 (2)	−0.0108 (19)	−0.0140 (19)	−0.0057 (18)
C12	0.043 (2)	0.038 (2)	0.051 (2)	−0.0127 (19)	−0.0055 (18)	−0.0070 (18)
C13	0.080 (3)	0.073 (3)	0.061 (3)	−0.033 (3)	−0.026 (2)	−0.008 (2)
C14	0.153 (5)	0.167 (6)	0.049 (3)	−0.115 (5)	−0.034 (3)	0.005 (3)
C15	0.160 (6)	0.143 (6)	0.069 (4)	−0.065 (5)	−0.007 (4)	−0.024 (4)
C16	0.165 (6)	0.123 (6)	0.108 (5)	−0.057 (5)	−0.010 (4)	−0.044 (4)
C17	0.053 (2)	0.061 (3)	0.065 (3)	−0.012 (2)	−0.022 (2)	−0.021 (2)
C18	0.062 (3)	0.060 (3)	0.060 (3)	−0.024 (2)	−0.018 (2)	−0.016 (2)
C19	0.086 (3)	0.062 (3)	0.097 (4)	−0.006 (3)	−0.037 (3)	−0.021 (3)
C20	0.091 (3)	0.094 (4)	0.065 (3)	−0.016 (3)	−0.031 (3)	−0.029 (3)
C21	0.048 (2)	0.051 (3)	0.066 (3)	−0.006 (2)	−0.020 (2)	−0.007 (2)
C22	0.045 (2)	0.047 (3)	0.064 (3)	−0.012 (2)	−0.008 (2)	−0.014 (2)
C23	0.041 (2)	0.039 (2)	0.054 (3)	−0.0058 (19)	−0.0110 (19)	−0.0098 (19)
C24	0.039 (2)	0.033 (2)	0.061 (3)	0.0009 (18)	−0.016 (2)	−0.0060 (19)
C25	0.043 (2)	0.039 (2)	0.070 (3)	−0.004 (2)	−0.020 (2)	−0.005 (2)
C26	0.055 (3)	0.057 (3)	0.079 (3)	−0.011 (2)	−0.031 (2)	−0.006 (2)
C27	0.048 (3)	0.060 (3)	0.090 (4)	−0.018 (2)	−0.022 (3)	−0.004 (3)
C28	0.048 (3)	0.057 (3)	0.078 (3)	−0.015 (2)	−0.015 (2)	0.001 (2)
C29	0.040 (2)	0.038 (2)	0.066 (3)	−0.0065 (19)	−0.013 (2)	−0.004 (2)
C30	0.045 (2)	0.049 (3)	0.064 (3)	−0.008 (2)	−0.006 (2)	−0.003 (2)
C31	0.050 (2)	0.036 (2)	0.060 (3)	−0.006 (2)	−0.012 (2)	−0.004 (2)
C32	0.044 (2)	0.043 (2)	0.067 (3)	−0.012 (2)	−0.011 (2)	−0.014 (2)
C33	0.080 (3)	0.082 (4)	0.062 (3)	−0.021 (3)	−0.005 (3)	−0.006 (3)
C34	0.163 (6)	0.124 (5)	0.058 (3)	−0.052 (5)	−0.001 (4)	−0.018 (3)
C35	0.190 (7)	0.125 (6)	0.086 (5)	−0.025 (6)	0.024 (5)	−0.009 (4)
C36	0.196 (9)	0.188 (9)	0.203 (8)	−0.089 (7)	0.074 (7)	−0.090 (7)
C37	0.063 (3)	0.053 (3)	0.057 (3)	−0.019 (2)	−0.010 (2)	−0.001 (2)
C38	0.055 (3)	0.063 (3)	0.052 (3)	−0.025 (2)	−0.005 (2)	−0.005 (2)
C39	0.064 (3)	0.074 (4)	0.080 (3)	−0.011 (3)	0.000 (3)	−0.009 (3)
C40	0.103 (4)	0.092 (4)	0.048 (3)	−0.034 (3)	−0.016 (3)	−0.009 (2)

### Geometric parameters (Å, °)

**Table:**

O1—C1	1.216 (4)	C17—C18	1.515 (5)
O2—C2	1.222 (4)	C17—H17A	0.9700
O3—C21	1.209 (4)	C17—H17B	0.9700
O4—C22	1.218 (4)	C18—H18A	0.9700
N1—C2	1.406 (4)	C18—H18B	0.9700
N1—C1	1.406 (4)	C19—H19A	0.9600
N1—C17	1.469 (4)	C19—H19B	0.9600
N2—C11	1.375 (4)	C19—H19C	0.9600
N2—C13	1.442 (5)	C20—H20A	0.9600
N2—H2	0.86 (2)	C20—H20B	0.9600
N3—C18	1.455 (5)	C20—H20C	0.9600
N3—C20	1.466 (4)	C21—C25	1.481 (5)
N3—C19	1.466 (5)	C22—C23	1.478 (5)
N4—C22	1.391 (5)	C23—C32	1.347 (5)
N4—C21	1.404 (5)	C23—C24	1.411 (5)
N4—C37	1.475 (4)	C24—C25	1.407 (5)
N5—C31	1.372 (5)	C24—C29	1.417 (5)
N5—C33	1.443 (5)	C25—C26	1.391 (5)
N5—H5	0.85 (2)	C26—C27	1.400 (5)
N6—C38	1.444 (5)	C26—H26	0.9300
N6—C40	1.463 (4)	C27—C28	1.366 (5)
N6—C39	1.468 (5)	C27—H27	0.9300
C1—C5	1.480 (5)	C28—C29	1.409 (5)
C2—C3	1.473 (5)	C28—H28	0.9300
C3—C12	1.351 (4)	C29—C30	1.410 (5)
C3—C4	1.412 (4)	C30—C31	1.382 (5)
C4—C5	1.401 (5)	C30—H30	0.9300
C4—C9	1.415 (5)	C31—C32	1.426 (5)
C5—C6	1.380 (5)	C32—H32	0.9300
C6—C7	1.400 (5)	C33—C34	1.467 (6)
C6—H6	0.9300	C33—H33A	0.9700
C7—C8	1.365 (5)	C33—H33B	0.9700
C7—H7	0.9300	C34—C35	1.514 (7)
C8—C9	1.408 (5)	C34—H34A	0.9700
C8—H8	0.9300	C34—H34B	0.9700
C9—C10	1.416 (5)	C35—C36	1.413 (8)
C10—C11	1.375 (5)	C35—H35A	0.9700
C10—H10	0.9300	C35—H35B	0.9700
C11—C12	1.419 (5)	C36—H36A	0.9600
C12—H12	0.9300	C36—H36B	0.9600
C13—C14	1.550 (6)	C36—H36C	0.9600
C13—H13A	0.9700	C37—C38	1.527 (5)
C13—H13B	0.9700	C37—H37A	0.9700
C14—C15	1.453 (7)	C37—H37B	0.9700
C14—H14A	0.9700	C38—H38A	0.9700
C14—H14B	0.9700	C38—H38B	0.9700
C15—C16	1.476 (7)	C39—H39A	0.9600
C15—H15A	0.9700	C39—H39B	0.9600
C15—H15B	0.9700	C39—H39C	0.9600
C16—H16A	0.9600	C40—H40A	0.9600
C16—H16B	0.9600	C40—H40B	0.9600
C16—H16C	0.9600	C40—H40C	0.9600
			
C2—N1—C1	124.9 (3)	N3—C19—H19C	109.5
C2—N1—C17	116.3 (3)	H19A—C19—H19C	109.5
C1—N1—C17	118.7 (3)	H19B—C19—H19C	109.5
C11—N2—C13	124.1 (3)	N3—C20—H20A	109.5
C11—N2—H2	113 (3)	N3—C20—H20B	109.5
C13—N2—H2	118 (3)	H20A—C20—H20B	109.5
C18—N3—C20	110.6 (3)	N3—C20—H20C	109.5
C18—N3—C19	108.1 (3)	H20A—C20—H20C	109.5
C20—N3—C19	109.0 (3)	H20B—C20—H20C	109.5
C22—N4—C21	125.5 (3)	O3—C21—N4	120.5 (4)
C22—N4—C37	115.9 (3)	O3—C21—C25	123.8 (4)
C21—N4—C37	118.4 (3)	N4—C21—C25	115.7 (4)
C31—N5—C33	122.4 (4)	O4—C22—N4	120.6 (4)
C31—N5—H5	113 (3)	O4—C22—C23	122.0 (4)
C33—N5—H5	120 (3)	N4—C22—C23	117.4 (4)
C38—N6—C40	111.6 (3)	C32—C23—C24	120.6 (3)
C38—N6—C39	109.5 (3)	C32—C23—C22	120.1 (4)
C40—N6—C39	108.7 (3)	C24—C23—C22	119.3 (4)
O1—C1—N1	120.0 (4)	C25—C24—C23	121.0 (3)
O1—C1—C5	123.7 (4)	C25—C24—C29	120.2 (4)
N1—C1—C5	116.3 (4)	C23—C24—C29	118.8 (4)
O2—C2—N1	120.0 (4)	C26—C25—C24	120.0 (4)
O2—C2—C3	123.2 (4)	C26—C25—C21	119.2 (4)
N1—C2—C3	116.9 (3)	C24—C25—C21	120.8 (4)
C12—C3—C4	120.1 (3)	C25—C26—C27	119.5 (4)
C12—C3—C2	119.6 (3)	C25—C26—H26	120.3
C4—C3—C2	120.3 (3)	C27—C26—H26	120.3
C5—C4—C3	120.7 (3)	C28—C27—C26	121.1 (4)
C5—C4—C9	120.5 (3)	C28—C27—H27	119.4
C3—C4—C9	118.7 (3)	C26—C27—H27	119.4
C6—C5—C4	120.3 (4)	C27—C28—C29	121.0 (4)
C6—C5—C1	118.8 (4)	C27—C28—H28	119.5
C4—C5—C1	120.9 (3)	C29—C28—H28	119.5
C5—C6—C7	119.4 (4)	C28—C29—C30	122.7 (4)
C5—C6—H6	120.3	C28—C29—C24	118.2 (4)
C7—C6—H6	120.3	C30—C29—C24	119.1 (4)
C8—C7—C6	120.7 (4)	C31—C30—C29	121.7 (4)
C8—C7—H7	119.6	C31—C30—H30	119.1
C6—C7—H7	119.6	C29—C30—H30	119.1
C7—C8—C9	121.5 (4)	N5—C31—C30	123.8 (4)
C7—C8—H8	119.2	N5—C31—C32	118.8 (4)
C9—C8—H8	119.2	C30—C31—C32	117.4 (4)
C8—C9—C4	117.5 (4)	C23—C32—C31	122.3 (4)
C8—C9—C10	123.0 (4)	C23—C32—H32	118.8
C4—C9—C10	119.6 (3)	C31—C32—H32	118.8
C11—C10—C9	121.1 (4)	N5—C33—C34	112.5 (4)
C11—C10—H10	119.5	N5—C33—H33A	109.1
C9—C10—H10	119.5	C34—C33—H33A	109.1
C10—C11—N2	124.3 (4)	N5—C33—H33B	109.1
C10—C11—C12	118.0 (4)	C34—C33—H33B	109.1
N2—C11—C12	117.7 (3)	H33A—C33—H33B	107.8
C3—C12—C11	122.6 (3)	C33—C34—C35	114.7 (5)
C3—C12—H12	118.7	C33—C34—H34A	108.6
C11—C12—H12	118.7	C35—C34—H34A	108.6
N2—C13—C14	109.9 (3)	C33—C34—H34B	108.6
N2—C13—H13A	109.7	C35—C34—H34B	108.6
C14—C13—H13A	109.7	H34A—C34—H34B	107.6
N2—C13—H13B	109.7	C36—C35—C34	114.9 (6)
C14—C13—H13B	109.7	C36—C35—H35A	108.5
H13A—C13—H13B	108.2	C34—C35—H35A	108.5
C15—C14—C13	116.4 (4)	C36—C35—H35B	108.5
C15—C14—H14A	108.2	C34—C35—H35B	108.5
C13—C14—H14A	108.2	H35A—C35—H35B	107.5
C15—C14—H14B	108.2	C35—C36—H36A	109.5
C13—C14—H14B	108.2	C35—C36—H36B	109.5
H14A—C14—H14B	107.3	H36A—C36—H36B	109.5
C14—C15—C16	116.1 (5)	C35—C36—H36C	109.5
C14—C15—H15A	108.3	H36A—C36—H36C	109.5
C16—C15—H15A	108.3	H36B—C36—H36C	109.5
C14—C15—H15B	108.3	N4—C37—C38	113.2 (3)
C16—C15—H15B	108.3	N4—C37—H37A	108.9
H15A—C15—H15B	107.4	C38—C37—H37A	108.9
C15—C16—H16A	109.5	N4—C37—H37B	108.9
C15—C16—H16B	109.5	C38—C37—H37B	108.9
H16A—C16—H16B	109.5	H37A—C37—H37B	107.8
C15—C16—H16C	109.5	N6—C38—C37	114.1 (3)
H16A—C16—H16C	109.5	N6—C38—H38A	108.7
H16B—C16—H16C	109.5	C37—C38—H38A	108.7
N1—C17—C18	113.6 (3)	N6—C38—H38B	108.7
N1—C17—H17A	108.9	C37—C38—H38B	108.7
C18—C17—H17A	108.9	H38A—C38—H38B	107.6
N1—C17—H17B	108.9	N6—C39—H39A	109.5
C18—C17—H17B	108.9	N6—C39—H39B	109.5
H17A—C17—H17B	107.7	H39A—C39—H39B	109.5
N3—C18—C17	114.7 (3)	N6—C39—H39C	109.5
N3—C18—H18A	108.6	H39A—C39—H39C	109.5
C17—C18—H18A	108.6	H39B—C39—H39C	109.5
N3—C18—H18B	108.6	N6—C40—H40A	109.5
C17—C18—H18B	108.6	N6—C40—H40B	109.5
H18A—C18—H18B	107.6	H40A—C40—H40B	109.5
N3—C19—H19A	109.5	N6—C40—H40C	109.5
N3—C19—H19B	109.5	H40A—C40—H40C	109.5
H19A—C19—H19B	109.5	H40B—C40—H40C	109.5
			
C2—N1—C1—O1	−175.7 (3)	C22—N4—C21—O3	−178.5 (4)
C17—N1—C1—O1	−0.1 (5)	C37—N4—C21—O3	−2.9 (6)
C2—N1—C1—C5	4.2 (5)	C22—N4—C21—C25	1.3 (5)
C17—N1—C1—C5	179.8 (3)	C37—N4—C21—C25	177.0 (3)
C1—N1—C2—O2	176.4 (3)	C21—N4—C22—O4	175.8 (4)
C17—N1—C2—O2	0.8 (5)	C37—N4—C22—O4	0.1 (5)
C1—N1—C2—C3	−4.5 (5)	C21—N4—C22—C23	−4.9 (5)
C17—N1—C2—C3	179.8 (3)	C37—N4—C22—C23	179.4 (3)
O2—C2—C3—C12	1.6 (5)	O4—C22—C23—C32	4.1 (6)
N1—C2—C3—C12	−177.4 (3)	N4—C22—C23—C32	−175.2 (3)
O2—C2—C3—C4	−178.8 (3)	O4—C22—C23—C24	−175.7 (4)
N1—C2—C3—C4	2.2 (5)	N4—C22—C23—C24	5.0 (5)
C12—C3—C4—C5	179.6 (3)	C32—C23—C24—C25	178.3 (3)
C2—C3—C4—C5	0.0 (5)	C22—C23—C24—C25	−1.9 (5)
C12—C3—C4—C9	−0.2 (5)	C32—C23—C24—C29	−2.7 (5)
C2—C3—C4—C9	−179.8 (3)	C22—C23—C24—C29	177.1 (3)
C3—C4—C5—C6	−179.8 (3)	C23—C24—C25—C26	179.1 (3)
C9—C4—C5—C6	0.0 (5)	C29—C24—C25—C26	0.1 (5)
C3—C4—C5—C1	−0.3 (5)	C23—C24—C25—C21	−1.8 (5)
C9—C4—C5—C1	179.5 (3)	C29—C24—C25—C21	179.2 (3)
O1—C1—C5—C6	−2.2 (6)	O3—C21—C25—C26	1.1 (6)
N1—C1—C5—C6	177.9 (3)	N4—C21—C25—C26	−178.7 (3)
O1—C1—C5—C4	178.3 (4)	O3—C21—C25—C24	−178.0 (4)
N1—C1—C5—C4	−1.6 (5)	N4—C21—C25—C24	2.1 (5)
C4—C5—C6—C7	−0.1 (6)	C24—C25—C26—C27	−1.0 (6)
C1—C5—C6—C7	−179.6 (3)	C21—C25—C26—C27	179.9 (4)
C5—C6—C7—C8	0.3 (6)	C25—C26—C27—C28	0.8 (6)
C6—C7—C8—C9	−0.4 (6)	C26—C27—C28—C29	0.3 (6)
C7—C8—C9—C4	0.2 (6)	C27—C28—C29—C30	178.5 (4)
C7—C8—C9—C10	−179.9 (4)	C27—C28—C29—C24	−1.1 (6)
C5—C4—C9—C8	−0.1 (5)	C25—C24—C29—C28	0.9 (5)
C3—C4—C9—C8	179.8 (3)	C23—C24—C29—C28	−178.1 (3)
C5—C4—C9—C10	−179.9 (3)	C25—C24—C29—C30	−178.8 (3)
C3—C4—C9—C10	−0.1 (5)	C23—C24—C29—C30	2.2 (5)
C8—C9—C10—C11	−179.1 (4)	C28—C29—C30—C31	−179.4 (4)
C4—C9—C10—C11	0.7 (5)	C24—C29—C30—C31	0.3 (6)
C9—C10—C11—N2	−178.0 (3)	C33—N5—C31—C30	−3.5 (6)
C9—C10—C11—C12	−1.1 (5)	C33—N5—C31—C32	178.4 (4)
C13—N2—C11—C10	−3.9 (6)	C29—C30—C31—N5	179.6 (4)
C13—N2—C11—C12	179.2 (4)	C29—C30—C31—C32	−2.3 (6)
C4—C3—C12—C11	−0.2 (5)	C24—C23—C32—C31	0.6 (6)
C2—C3—C12—C11	179.4 (3)	C22—C23—C32—C31	−179.2 (3)
C10—C11—C12—C3	0.8 (5)	N5—C31—C32—C23	−179.9 (4)
N2—C11—C12—C3	178.0 (3)	C30—C31—C32—C23	1.9 (6)
C11—N2—C13—C14	164.1 (4)	C31—N5—C33—C34	176.3 (4)
N2—C13—C14—C15	−170.6 (5)	N5—C33—C34—C35	−179.2 (5)
C13—C14—C15—C16	61.5 (8)	C33—C34—C35—C36	93.2 (8)
C2—N1—C17—C18	−79.6 (4)	C22—N4—C37—C38	−77.6 (4)
C1—N1—C17—C18	104.4 (4)	C21—N4—C37—C38	106.3 (4)
C20—N3—C18—C17	−68.8 (4)	C40—N6—C38—C37	−68.5 (4)
C19—N3—C18—C17	172.0 (3)	C39—N6—C38—C37	171.0 (3)
N1—C17—C18—N3	−69.1 (4)	N4—C37—C38—N6	−70.1 (4)

### Hydrogen-bond geometry (Å, °)

**Table:**

*D*—H···*A*	*D*—H	H···*A*	*D*···*A*	*D*—H···*A*
N2—H2···N6	0.86 (2)	2.33 (2)	3.176 (5)	173 (3)
N5—H5···N3	0.85 (2)	2.37 (2)	3.220 (5)	172 (4)
